# Another benefit of regular sleep

**DOI:** 10.7554/eLife.94131

**Published:** 2023-12-01

**Authors:** Tianyi Huang

**Affiliations:** 1 Channing Division of Network Medicine, Brigham and Women’s Hospital and Harvard Medical School Boston United States; 2 Division of Sleep Medicine, Harvard Medical School Boston United States

**Keywords:** sleep, sleep regularity, UK Biobank, circadian rhythms, mortality, actigraphy, None

## Abstract

A large observational study has found that irregular sleep-wake patterns are associated with a higher risk of overall mortality, and also mortality from cancers and cardiovascular disease.

**Related research article** Cribb L, Sha R, Yiallourou S, Grima NA, Cavuoto M, Baril AA, Pase MP. 2023. Sleep regularity and mortality: a prospective analysis in the UK Biobank. *eLife* **12**:RP88359. doi: 10.7554/eLife.88359.

Benjamin Franklin’s famous quotation – “Early to bed and early to rise makes a man healthy, wealthy, and wise” – underscores the importance of sleep-wake patterns to our health and well-being. Of all the phenomena that are influenced by the 24 hour light-dark cycle, the sleep-wake cycle is the best known, but many other biological processes central to health are also influenced. However, empirical evidence about the impact that disruptions to sleep-wake patterns have on human health remains sparse and is mostly limited to studies on shift workers, despite an increase in other forms of disruption (notably higher levels of artificial light at night and increased use of mobile media in bed).

In recent years, an increasing number of studies have looked at the impact of sleep regularity (and irregularity) on aspects of health besides sleep quantity and quality, often made possible by the availability of wearable technology that is able to monitor rest-activity cycles in large numbers of people. Studies have found evidence that sleep irregularity is associated with a higher incidence of obesity (Patel et al., 2014), depression (Fang et al., 2021), cardiovascular disease (Huang et al., 2020) and other chronic disease outcomes.

Now, in eLife, Matthew Pase, Andree-Ann Baril and colleagues – including Lachlan Cribb, Ramon Sha and Stephanie Yiallourou, all of Monash University, as joint first authors – go one step further by linking sleep regularity with all-cause and cause-specific mortality in a large UK population (Cribb et al., 2023). Between 2013 and 2015, a total of more than 106,000 participants in the UK wore a watch-like device called an accelerometer over seven consecutive days. Based on the signals from this device it was possible to tell when the participants were asleep, sedentary, or active. Cribb et al. then derived a novel metric called the Sleep Regularity Index (SRI) to quantify day-to-day consistency in sleep-wake schedules. This metric is an estimate of how likely it is that a person is in the same state (that is, either awake or asleep) at any two time points 24 hours apart, with higher SRI values indicating higher sleep regularity. The researchers also noted how many of the participants subsequently died (via data from the UK national death register).

Over a median follow-up of 7.1 years, some 3,010 participants died, with 1,701 of these deaths being due to cancers and 616 being due to cardiovascular disease. Compared with those who had a medium SRI value, the top 5% (that is, the most regular sleepers) had 10% lower all-cause mortality, and the bottom 5% (that is, the most irregular sleepers) had 53% higher mortality. The association appeared nonlinear, with stronger associations observed for the increased mortality with more irregular sleep. Similar nonlinear patterns were observed when Cribb et al. examined cancer mortality and cardiovascular disease mortality separately.

The work by Cribb et al. is consistent with recent studies on sleep regularity and mortality from the US and Japan, although these findings are based on a smaller sample size (Chung et al., 2023a) or on self-reported sleep regularity (Omichi et al., 2022). Other studies found that sleep regularity was a stronger predictor for mortality than sleep duration or sleep apnea (Chung et al., 2023b; Windred et al., 2023). Interestingly, a genome-wide association study for sleep traits (Jones et al., 2019) reported a heritability estimate of 2.8% for sleep regularity, compared with 19.0% for sleep duration and 22.3% for fragmented sleep: this suggests that it may be easier to modify sleep regularity via interventions than it would be to modify other sleep traits because sleep regularity is less genetically determined.

Promoting sleep regularity is not a new idea in the area of ‘sleep hygiene’, and encouraging regular sleep schedules is also an essential component in cognitive behavioral therapy for insomnia (Sletten et al., 2023). From the behavioral perspective, promoting sleep regularity may also enhance the time-of-day effect of other behaviors, such as time-restricted eating and exercise. However, promoting sleep regularity for broader health promotion and disease prevention has not been tested in intervention studies and requires additional evidence from future investigations.

In summary, the study by Cribb et al. adds to the emerging evidence for the potential benefits of sleep regularity on chronic disease risk and mortality. The findings, in conjunction with prior evidence, provide a strong foundation to answer the following question: would public health interventions to improve sleep regularity causally lead to reductions in chronic disease and premature deaths at the population level?
